# Supplementary material for: Development of a computer-aided design software for the quantitative evaluation of aesthetic damage
Source: PLoS One. 2019 Dec 18;14(12):e0226322. doi: 10.1371/journal.pone.0226322 (PMC6919621; doi:10.1371/journal.pone.0226322)
Supplement: S1 Code site — 1. (ZIP) [file pone.0226322.s001.zip › [EESC_jr][Projeto]Relatorio_07_21/reports/templates_zip/gentelella-master/vendors/morris.js/examples/non-date.html]

# Formatting Non-date Arbitrary X-axis

```
var day_data = [
  {"elapsed": "I", "value": 34},
  {"elapsed": "II", "value": 24},
  {"elapsed": "III", "value": 3},
  {"elapsed": "IV", "value": 12},
  {"elapsed": "V", "value": 13},
  {"elapsed": "VI", "value": 22},
  {"elapsed": "VII", "value": 5},
  {"elapsed": "VIII", "value": 26},
  {"elapsed": "IX", "value": 12},
  {"elapsed": "X", "value": 19}
];
Morris.Line({
  element: 'graph',
  data: day_data,
  xkey: 'elapsed',
  ykeys: ['value'],
  labels: ['value'],
  parseTime: false
});
```
